# Preferences of Female Triathletes and Their Coaches for Education on REDs: A Qualitative Study

**DOI:** 10.1002/ejsc.70240

**Published:** 2026-08-14

**Authors:** Penny Slater, Naroa Etxebarria, Michelle Minehan, Angie Fearon

**Affiliations:** ^1^ University of Canberra Research Institute for Sport and Exercise (UCRISE) Canberra Australian Capital Territory Australia

## Abstract

Participation in triathlon may increase athletes' exposure to risk factors associated with Relative Energy Deficiency in Sport (REDs); however, effective education could enhance understanding and help mitigate risk. The views of experienced triathletes and coaches need to be considered when developing education resources aimed at improving identification and management of the syndrome. This qualitative study aimed to understand how female athletes and their coaches engaged with REDs resources, their preferences for education delivery and challenges to engaging with educational material. Twenty semi‐structured interviews were conducted with 12 female triathletes aged 25–45 years and 8 coaches aged 29–62 years. Participants included amateur (*n* = 8) and elite (*n* = 4) triathletes. Reflexive thematic analysis was utilised to construct five themes: 1. Understanding of REDs; 2. Experiences associated with REDs; 3. Engagement with educational resources; 4. Opportunities for REDs education and 5. Challenges to engaging with REDs education. Themes were mapped to the capability, opportunity, motivation‐behaviour (COM‐B) model to identify factors likely to influence engagement with a REDs education resource for female triathletes and their coaches. Both athletes and coaches voiced strong motivation to engage with REDs education and an interest in learning more about REDs. However, future engagement is likely to be influenced by the entrenched culture that encourages leanness in triathlon, and resistance to modifying training practices. Our analysis identifies the opportunity to improve female triathletes' understanding of REDs, by using podcasts that combine expert led content with lived‐experience narratives from other triathletes.

## Highlights

- Female triathletes and their coaches are strongly motivated to learn more about REDs and seek education resources on the topic.
- Future educational resources for triathletes and coaches should focus on addressing tensions that exist in the entrenched leanness culture and normalisation of REDs symptoms within triathlon.
- Female triathletes strongly favour the use of podcasts for consuming educational material whilst coaches favour practical workshops and online modules.
- Both female triathletes and coaches wish to learn from experts and engage with lived‐experienced narratives.

## 1 Introduction

Relative Energy Deficiency in Sport (REDs) is a complex syndrome caused by prolonged and/or severe low energy availability (LEA) (Mountjoy et al. 2023). LEA occurs when dietary intake is insufficient to meet the demands of exercise energy expenditure, as well as to sustain normal physiological functions (Loucks et al. 1998). Prolonged LEA can lead to adverse health outcomes, including impairments in energy metabolism, reproductive and musculoskeletal health, and immune function (Gowers et al. 2025; Jeppesen et al. 2024; DeJong Lempke et al. 2025). Negative consequences to athletic performance are also evident in athletes experiencing REDs, as they are at higher risk of injury and illness, as well as reduced recovery and adaptation to training (Mountjoy et al. 2023).

Triathlon consists of three different individual sports (swim, bike, run), resulting in high training volume. In female triathletes, the training hours during competition season has been reported to be between 7 and 18 h a week in amateur athletes and 17–27 h a week in elite level triathletes (Slater et al. 2026). High training volumes increase the likelihood of LEA if athletes do not manage their nutrition intake accordingly. Research indicates that triathletes often show signs of energy conservation, reduced energy availability and lower bone mineral density scores across an athletic season (Jesus et al. 2022). Across all sports, the reported prevalence of REDs is similar for male and female athletes (Gallant et al. 2025). However, some research indicates that female triathletes are disproportionately affected by symptoms associated with REDs. For example, Crunkhorn et al. (2022) found the rate of bone stress injuries to be 2.7 times higher in female triathletes than males (Crunkhorn et al. 2022). Jesus et al. (2022) found that over a preparatory phase and competition season fewer female triathletes than males reached optimal energy availability (Jesus et al. 2022). Although male athletes have been disproportionately represented in research related to REDs, gender differences in manifestations and causes of REDs have been reported (Ruscheck et al. 2025). Female triathletes are displaying higher rates of symptoms associated with REDs than male triathletes, indicating they should be a target audience for future education interventions on REDs.

A number of studies have investigated athlete perspectives of REDs, specifically in male (4) and female (8) lightweight rowers, endurance runners (10 female, 2 male) and adolescent female team sport athletes (13) (Gillbanks et al. 2022a; Langbein et al. 2021; O'Donnell et al. 2023). These studies collectively identify the need to improve awareness and understanding of LEA and REDs. Our own survey of female triathletes and coaches identified that understanding of REDs was limited: athletes had low confidence in seeking help and managing symptoms related to REDs, and had low awareness of available resources to identify, prevent, and/or manage REDs (Slater et al. 2026). Survey respondents expressed strong interest in resources that were specific to female triathletes. Educational interventions have traditionally adopted a didactic approach with limited incorporation of athlete and coaches' perspectives into their design and delivery. Involving athletes and coaches in the development and translation of education interventions may facilitate wider dissemination and knowledge retention.

Translational research is a pragmatic research approach that focusses on the practical application of scientific findings (Allan et al. 2024). Education is most effective when targeted to a specific audience (Jones and McLean 2012). There are some excellent quality resources that address REDs (Australian Institute of Sport 2019; ProjectREDs 2020; REDsinSport 2019). However, there are few triathlon‐specific resources available that have been designed with input from triathletes and coaches (Hamer et al. 2021). Understanding female triathletes' and coaches' perspectives of REDs, alongside their previous educational experiences, is essential to inform the development of practical and targeted educational tools that will increase the likelihood of engagement, knowledge retention and behaviour change (Holt et al. 2018). Pedagogy can best be applied when the specific learning preferences of audiences are considered in the design stage (Damşa and de Lange 2019). To successfully develop REDs educational resources tailored to the female triathlon population, it is essential to understand this cohort's knowledge status, learning preferences and challenges to engaging with education.

The aim of this study was to understand how female triathletes and their coaches access and utilise REDs resources, their preferences for access to educational content delivery and the challenges they face when engaging with REDs education.

## 2 Methodology

### 2.1 Research Philosophy

This study was underpinned by a pragmatic research philosophy aiming to generate practical knowledge (Ponterotto 2005). A pragmatic approach allows for understanding context and ‘real world’ processes that affect participants, creating meaningful and actionable insights (Creswell and Creswell 2003). Pragmatism aligns closely with the goal to develop REDs education that considers the views, experiences and preferences of female athletes and their coaches.

### 2.2 Study Design

Semi‐structured interviews were conducted with female triathletes and coaches of female triathletes. A semi‐structured interview guide was developed by the research team in alignment with the research aims of the study. Interview questions focused on athlete and coach backgrounds (characterisation), current knowledge and experience of REDs, preferences for delivery style, previous education experiences and perception of culture relevant to REDs within triathlon (Supporting Information S1). To gain experience the interviewer conducted mock interviews with the other authors and adjusted interview style based on feedback. The interview guide was piloted on two participants who were not included in the dataset to test for clarity of questions, interviewer technique and interview flow. Upon receiving feedback, the research team amended four questions to improve clarity and flow of the interview. All interviews were conducted by PS using an online platform, Microsoft teams (version 4.2.4.0). All participants were in a quiet office space or room at home whilst the interviews were conducted. Written informed consent was provided by all participants. Ethical approval was provided by the University of Canberra Ethics Committee number (HREC‐13719). This research is reported according to the Consolidated Criteria for Reporting Qualitative Research (COREQ) (Tong et al. 2007) (Supporting Information S1).

### 2.3 Sample Size Estimation and Justification

Participants were recruited via social media, and advertisements in triathlon clubs and professional association newsletters. The inclusion criteria for athletes were any female triathlete over the age of 18 years currently competing in triathlon, for coaches it was any triathlon coach over the age of 18 who currently coached female triathletes. Twenty‐five female triathletes and fifteen coaches of female triathletes expressed interest in being interviewed. Interviews were scheduled according to order of expressing interest. Twelve athletes and eight coaches were interviewed over a 6‐month period. The final number of interviews was guided by Malterud's concept of sufficient information power (Malterud et al. 2016). A preliminary analysis was undertaken after eight athletes and six coaches were interviewed. A sufficient depth of data was established, however, an additional four athletes and two coaches were interviewed to ensure that a sufficient depth of information power had been reached (Malterud et al. 2016).

### 2.4 Data Analysis

Data were analysed guided by reflexive thematic analysis (Braun and Clarke 2019). Braun and Clarke (2022) propose that coding can be flexible to ensure that researchers engage with data thoughtfully and are reflexive during the analytical process (Braun and Clarke 2022). As such, the codes were developed inductively into a deductive framework, developed and shaped by the research aims and questions (Braun and Clarke 2022). Prior to analysis, the recordings and transcripts were deidentified. To ensure deep immersion in the data, PS read and reread the transcripts several times and developed preliminary codes based on these readings. In the second phase, the rest of the research team read the transcripts and provided feedback and adjustments to codes. In the third phase, the research team reread transcripts to ensure that codes were consistent across the transcripts, and constructed preliminary themes (Braun and Clarke 2019). The fourth phase involved themes and codes being reviewed and refined, interrogating again whether they provided meaningful interpretations of the data and addressed the research aims. This was facilitated by a whiteboard session and discussions across the research team. In this stage, codes were confirmed, and themes were fully defined ensuring that the themes were consistent across the data. In the fifth phase, PS used qualitative data analysis software (ATLAS.ti, version 23.1.1, 2023) to code the transcripts for auditability and identify data extracts. The sixth and final phase of the analysis involved writing the report that was woven throughout the entire analytical process completing the recursive nature of the reflexive thematic analysis (Braun and Clarke 2019).

Themes were mapped to the COM‐B (Capability, Opportunity, Motivation‐Behaviour) model which posits capability, opportunity and motivation must be present for behaviour change to occur (Michie et al. 2011). The COM‐B model was used to critically assess factors likely to influence engagement with a REDs education resource for female triathletes and their coaches. Capability refers to triathletes and coaches' knowledge and psychological skills to engage with REDs education for prevention and management. Opportunity refers to the factors that may facilitate or hinder the implementation of evidence‐based guidelines or education content available to manage/avoid REDs. Motivation involves identifying internal drivers or beliefs that influence engagement in REDs resources. Together, capability, opportunity and motivation may shape the behaviour of athletes and their coaches.

### 2.5 Reflexivity

The primary researcher is a female athlete and triathlon coach, deeply embedded in the triathlon landscape within Australia. Co‐researchers are a female exercise physiologist, dietitian and physiotherapist with decades of experience in elite sport and clinical settings. The research team have either directly experienced REDs or managed athletes with REDs. No researcher had any significant prior relationship with participants. The primary researcher engaged in reflexivity throughout the entire process, keeping study notes and memos (Olmos‐Vega et al. 2023). All authors were involved in the thematic analysis process bringing diverse professional and lived experience to the interpretation of the data (Braun and Clarke 2022). Through discussions and reflexive engagement with the dataset, the research team considered alternative interpretations and refined themes to ensure they provided meaningful and accurate accounts grounded in participants' experiences.

## 3 Results

Twenty interviews were conducted with female triathletes (*n* = 12) and coaches of female triathletes (*n* = 8). Seven coaches were male (*n* = 7) and one was female (*n* = 1). Athletes were aged between 25 and 45 years and coaches aged between 29 and 62 years. Athletes were from Australia (*n* = 6), New Zealand (*n* = 1), the United States of America (*n* = 3), Netherlands (*n* = 1) and the United Kingdom (*n* = 1). Coaches were from Australia (*n* = 6), New Zealand (*n* = 1) and the United Kingdom (*n* = 1). The focus for most coaches and athletes was long distance triathlon with coach experience ranging from 4 to 35 years and athletes experience ranging from 4 to 7 years. Within the athlete cohort, there was a mix of elite (*n* = 4) and amateur (*n* = 8) with their elite/amateur status being determined by their national triathlon bodies. Five of the coaches were, at the time of the study, coaching at least one elite athlete. Interviews ranged from 25 to 60 min in duration.

### 3.1 Thematic Analysis Overview

Five themes were constructed: 1. Understanding of REDs, 2. Experiences associated with REDs, 3. Engagement with Educational Resources, 4. Opportunities for REDs education and 5. Challenges to engaging with REDs education.

### 3.2 Understanding of REDs

Understanding of REDs was limited in this sample, although participants were able to recognise it as a mismatch between energy intake and energy expenditure. As coach 8 explained, ‘*So basically, energy deficiencies, just the energy that you're taking into your body is not matching your output. So basically your training output, it's higher than the energy you're getting in from your fuel’*. Some described the syndrome as a general fatigue or overtraining syndrome. For example, ‘I just kind of took it as a little bit of like overtraining. So going to the point of overtraining and under fuelling.’ (Athlete 6, amateur). Four athletes and three coaches articulated one or more health or performance consequences associated with REDs and only one athlete and two coaches were able to identify all components of REDs including, energy deficit, physiological, psychological and performance consequences.

### 3.3 Experiences Associated With REDs

Two athletes in this study reported a diagnosis of REDs previously, emphasising the detrimental effect on their health and well‐being. Other athletes reported symptoms which are often associated with REDs including bone stress injuries, amenorrhoea and burnout. For example, Athlete 1 (*elite*) reported two bone stress injuries in their career. They now attribute this to REDs *‘I saw a dietitian and yeah I was under‐fuelling, We did a fair bit of work on timing of fuelling, I definitely think that REDs was part of that then’.* Coaches also reported working with athletes who had been diagnosed with REDs, particularly identifying young females as a high‐risk group. Coach 8 reported, ‘*majority of athletes who I coach, yes I would see REDs symptoms, and particularly if I get a new athlete in and particularly a young female athlete in I would say it often I would see it (LEA and REDs‐related symptoms) in men and women, but definitely particularly young women, I would see it a lot more’*.

Both athletes and coaches voiced how firsthand experience with symptoms within the REDs model strengthened their understanding of the syndrome, especially when they experienced positive changes after addressing LEA. For example, Athlete 5 (*elite*) explained, ‘*and then obviously then when my performance and my training and my health started turning around, I really did then believe in the process I bought into’.* Similarly, Athlete 11 (*amateur*), described *‘being able to function for the rest of the day versus just like being a zombie on the couch for the remainder of the day each time’.* Coaches described the performance‐related changes they observed in athletes when they had recovered from REDs and the ‘buy in’ that it created for them to continue to ensure athletes were remaining healthy, *‘you know, one of them messaged me the other day and she said she had a period for the first time in about 2 years. And actually she's just going better and better with her Ironman training* *’*. (Coach 3).

### 3.4 Engagement With Educational Resources

Athletes typically accessed information about REDs and other triathlon‐related content such as nutrition and training guidelines through specific searches on the internet, podcasts and social media. Athletes described looking for easy answers in quickly accessible forms. For example, searching ‘*What is REDs*?’ and ‘*symptoms of overtraining’* on platforms such as Google and Instagram. They described looking for resources that were digestible for an athlete ‘*that makes you actually want to, like, buy in and learn*’ (Athlete 5, elite). Athletes mostly spoke about accessing information about REDs via social media and podcasts as they were ‘time poor’, ‘*when you think about triathlon you end up training for many hours by yourself, so for me it's (education resources) in the form of podcasts*' (Athlete 12, amateur). Athletes expressed frustration trying to navigate some of the conflicting and complex information available about REDs explaining that ‘*it's just confusing and a lot of work because I just keep getting all these conflicting answers and so trying to kind of find exactly where and what is the consequence, it's been a lot harder than I expected to get it right*’ (Athlete 2, amateur).

Coaches also utilised the internet and social media but sought additional research‐informed resources with six of the eight coaches accessing journal articles. Coaches spoke about the complexity of understanding REDs and how it was challenging to find ‘*information on such a potentially diverse topic, where it can affect everybody differently’* (Coach 7). Coaches also spoke about being aware of their, *‘scope of practice and making sure that I'm sticking within the boundaries.’* (Coach 4). They expressed a need for resources to help them identify athletes who, *‘had a red flag against them as being candidates for REDs’* (Coach 3), so they could connect them with appropriate support like a sports doctor and dietitian. One coach expressed frustration that his triathlon organising body provided limited resources about REDs when it was a known problem among athletes, *‘I don't even know if (National Sporting Body) really try and educate us much about it. And I know it's been a bit of an issue with the (country) elite female triathletes recently’* (Coach 3).

### 3.5 Opportunities and Preferences for REDs Education

Both athletes and coaches expressed strong interest in improving their understanding of REDs. Athletes expressed the need to take responsibility for learning about REDs themselves, as they perceived their coach to have limited knowledge of the topic. Athlete 9 *(amateur)* explained, *‘I've never been given information by a coach on REDs. And the conversation has always been initiated by me’.* Athletes favoured digital content, such as audio formats and social media, citing ease of access and listening to podcasts and other audio formats whilst training as reasons for this. For example, Athlete 2 *(amateur)* voiced, *‘podcasts are the best for me as they are much easier if I'm going out for a long run’.* Similarly, Athlete 8 *(elite)* explained that audio books and podcasts were convenient when, ‘*I'm driving or when I'm on like a longer run that doesn't have intervals and I can just put it on and kind of have it on sort of in the background’.* Overall, the majority of athletes indicated they would prefer to consume content on REDs in the format of a podcast over any other delivery mediums.

Coaches expressed a preference workshops or practical tutorials. They valued opportunities to engage in face‐to‐face discussion. For example, Coach 5 explained, ‘*I think like tutorials or like workshops they are a really good way to explore REDs because then you get the opportunity for question answer time’*. Other coaches explained they felt that workshops offered a good opportunity to cover both theory and practice, *‘simple straight facts that help us out with what we need to do to get the information and message across to the athletes’* (Coach 2.) Some coaches explained feeling uncertain about how to initiate conversations relevant to aspects of LEA and REDs, *‘I think it's maybe like, but it's maybe tricky topic for me to discuss with them as a male coach and I'm sort of aware of that as well.’* (Coach 5). They identified that a workshop might be a more suitable option for *‘learning practically’* (Coach 2) about how to navigate such conversations.

Whilst coaches and athletes differed in their preferred mode of content delivery, both groups strongly expressed a desire for *‘real’ (Athlete 8, elite)* experiences from athletes supported with research‐backed information. It was important that content was *‘super relatable to an athlete and particularly younger athletes’* (Coach 8). Both coaches and athletes shared a common desire for the resource to include a forum where they could ask topic‐specific questions and receive answers from an expert. *‘I think having someone to turn to (to ask questions) like a nutritionist or sports doctor or someone who knows the science is who I would want to ask (about REDs)’* (Athlete 4, amateur). Coach 7 described their desire to interact with an expert *‘I want someone (an expert) who I can probe and ask things to that I can try out on a case study’*. Effective engagement techniques depended heavily on the delivery with both coaches and athletes favouring a *‘passionate, energized’* (Athlete 8, elite) presenter who was able to provide context specific education.

### 3.6 Challenges to Engaging With REDs Education

Although coaches and athletes were generally enthusiastic and supportive of learning more about REDs, there were issues that challenged the likelihood of future engagement with REDs educational resources. Some athletes expressed reticence to learn more about REDs or seek support due to fears of being excluded from training or competition. Athlete 8 *(elite)* describes other athletes in their triathlon community experiencing symptoms in the REDs model ‘no one wanted to admit it (they were experiencing REDs or LEA).. it was more like harden the f**k up mentality of training and racing’. There was also tension between being concerned about LEA and symptoms in the REDs model and accepting it as a ‘normal’ or ‘necessary’ part of being a triathlete. There was widespread acknowledgement that amenorrhoea has been and at times still is perceived as ‘normal' for female athletes, ‘*I think it (absence of menstrual period) used to be really almost like celebrated, almost like a badge of honour in a sense’* (Athlete 5, elite). Athlete 3 *(amateur)* explained that she equated ‘*if your body fat is so low you won't get your period’ to ‘being like ‘you're in really good shape, right’.* Coaches echoed this, *‘I would spend an hour each week talking to various female athletes on the topic (amenorrhoea). If not more so and I still can't get through to them’* (Coach 8).

Participants also identified that triathletes have a mentality of needing to ‘push through’ fatigue and injury. As Athlete 5 (*elite*) explained, ‘*so it's like normal that I feel fatigued, but what is an OK level of fatigue and like what isn't is sometimes really hard to judge yourself.’* Similarly, Athlete 9 *(amateur)* explained that it was normal to dismiss symptoms like fatigue, *‘myself included, we'll just dismiss kind of what they think is not really anything like whether it's just a bit of fatigue or whatever and just think “ohh well I've been training really hard’”*.

Despite growing awareness of the risks associated with under‐fuelling, athletes and coaches identified that a culture that encourages leanness acts as a deterrent to engaging with REDs education. Athletes described feeling pressure to be lean, and the perception that lean bodies were valued within the sport. Athlete 3 *(amateur)* voiced that, ‘*we're all striving for low body fat and to hear the fact that it's negative was, like, mind‐blowing, mind‐blowing to me’.* Athlete 7 (*elite*) explained, *‘I hear so many people talking about just wanting to get lean and just needing to be lean and not the healthy way of how they're gonna get there I suppose or why they would need to do that in the first place’*. Athlete 5 *(elite)* recounted being sent home with a stress injury but received conflicting messages about how to recover, but also to stay lean, ‘*they sent me home and the dietitian dropped me at the airport and as he was dropping me off, he was like now just try not to put on too much weight while you're having time off all, you'll never come back and it's like that was my first experience with the stressy’*.

Coaches also recognised the tension between minimising the risk of REDs and the perception that being lightweight is perceived as a performance indicator in triathlon. Coach 1 explained, ‘*I think people still idolize the thin. And you know most of the time I'm going to say most of the elite performers or Olympic people in triathlon specifically do have a slender body type’.* Coach 7 reinforced this, *‘100% body weight is emphasised and unfortunately, at probably the worst age group, to emphasize it as well, because they do, the youth coaches in particular, but you can still hear those kind of conversations a kilo here or kilo there you know to make race weight’*. These tensions could challenge motivation to engage with REDs education.

### 3.7 Practical Implications for the Implementation of REDs Education

Table 1 summarises how findings were mapped to the COM‐B model to identify strategies for successful design of a REDs educational resource for female triathletes.

**TABLE 1:** Identification of factors likely to influence engagement with a REDs education resource for female triathletes and their coaches.

COM‐B component	Categorisation of COM‐B components	Theme	Interaction between theme and COM‐B components
Capability *Psychological and physical capacity to engage in the activity‐ includes having the necessary knowledge and skills*	Physical *Practical skills, physical means*	Theme 3: Engagement with educational resources	Both athletes and coaches have physical means and practical skills to access freely available internet resources including podcasts that can be consumed within daily schedules.
	Psychological *Knowledge, reasoning, cognitive skills*	Theme 1: Understanding of REDs	Broad awareness of REDs with some limitations in understanding of the full range of impacts and capability to acquire knowledge within athletes and coaches.
Opportunity *Brain processes that energise and direct behaviour‐ includes habitual processes, emotional responding, analytical decision‐making*	Social *Is behaviour socially supported?*	Theme 4: Opportunities for REDs education	Podcasts are a commonplace and socially accepted education tool.
		Theme 5: Challenges to engaging with REDs education.	There is entrenched normalisation of REDs and LEA symptoms which may challenge divergent narratives in an education resource.
	Physical Are *resources available?*	Theme 3: Engagement with educational resources	Some existing resources available but there is a desire for more triathlon‐specific resources.
Motivation *Factors that lie outside the individual that make the behaviour possible or prompt it*	Automatic *Emotions, biases, reinforcements*	Theme 4: Opportunities for REDs education	Desire for education resource to be drawn from athlete/coach lived experience, with natural storytelling.
	Reflective *Beliefs, identity, intentions, goals*	Theme 5: Challenges to engaging with REDs education.	Tension between tightly held beliefs/identity of performance requiring leanness and the consequences of REDs symptoms.

## 4 Discussion

This study investigated the experiences and perspectives of female triathletes and their coaches, in order to inform the development of educational resources aimed at preventing REDs. This is important, because triathletes are vulnerable to symptoms within the conceptual model of REDs (Langa et al. 2025; Witkoś et al. 2023). The International Olympic Committee has called for the development of education resources to support prevention and management of REDs (Mountjoy et al. 2023). We identified that athletes and coaches were interested in better understanding REDs, and most likely to engage with podcasts (athletes) and online modules or workshops (coaches). However, normalisation of REDs symptoms, ‘push‐through’ mentality and a prevailing leanness culture challenge the likelihood that athletes and coaches will engage with REDs education. Currently, availability of triathlon‐specific REDs educational programs is limited (DeJong Lempke et al. 2025; Hamer et al. 2021). Some research has investigated education preference and the effectiveness of different educational approaches in triathlon and endurance athletes (Fahrenholtz et al. 2023; Roche et al. 2024; Solly et al. 2023). In a cross‐sectional study on nutrition education preferences among, rowing, cycling and athletics athletes in New Zealand and Australia, the most highly rated teaching techniques were real‐life examples, visual content and discussion with facilitators (Solly et al. 2023). Fahrenholzt conducted a 16‐week intervention for triathletes, runners, orienteers, cross country skiers and biathletes at risk of REDs, utilising lectures and one‐on‐one counselling sessions. REDs knowledge improved, whilst behaviour change for dietary intake was weak at the conclusion of the intervention (Fahrenholtz et al. 2023). Whilst these studies provide some insight into endurance athletes' learning preferences, further consultation with triathletes and coaches is needed to develop specific resources that are most likely to engage and influence female triathletes and coaches.

Coaches and athletes in our study supported the development of REDs education, specifically for female athletes. However, they had different preferences for content delivery. Triathletes favoured podcasts, whilst coaches preferred formal workshop/module approaches. This disparity between the groups, suggests that separate, but complementary, resources may need to be developed to cater for the specific preferences of learners. This approach could be essential to improve knowledge retention and engagement (Damşa and de Lange 2019). Surveys of Australian and New Zealand athletes from mixed sports ranked podcasts and social media delivery well below group education and one‐on‐one consultations with dietitians (Solly et al. 2023; Trakman et al. 2018). This might reflect differences in the way triathlon functions compared to other sports, particularly the high training load and the predominantly independent nature of training. Podcasts provide a good opportunity for female triathletes to engage with REDs education as they can be accessed easily and flexibly within demanding training schedules. However, it is also important to acknowledge that the diversity of perspectives presented in current podcasts may introduce variability in messaging that some athletes could find confusing. Podcasts also provide opportunity to engage with deeper and more detailed content compared to short‐form social media communication (Robins et al. 2024). The use of podcasts as an education tool has been beneficial in other learning environments (Savall Ceres and Villafán Amezcua 2025). Learners value the portability and efficiency of the format and medical students demonstrated equivalent knowledge retention when using podcasts compared to traditional education methods (Kelly et al. 2022). Podcasts are an increasingly popular and flexible education medium (Drew 2017). However, educational podcasts should be developed by experts in the topic to ensure the resource is valid and successful (Leite et al. 2022). Whether podcasts support behaviour change in triathletes remains to be investigated. These findings clearly identify podcasts as the preferred channel for the triathlete participants. However, further investigation is needed to confirm these preferences, determine the appropriate format and content and mitigate challenges such as conflicting information.AUTHOR: Please check whether the edits made in the sentence beginning with “However, further investigation is needed to …” are correct.

Coaches and athletes strongly voiced preference for an education platform in which they had the ability to ask questions of an expert. Drew (2017) characterises podcasts as ‘quick burst’, ‘narrative’ and ‘chat show’. These styles all allow opportunity to combine athlete, coach and health expert voices. However, to fully satisfy athletes, podcasts should be connected to expert‐moderated discussion forums and/or question‐answer platforms. The use of a question‐answer platform has the potential to create ‘buy in’ and learner autonomy which in turn increases the likelihood of engagement and retention of knowledge (Major et al. 2021). Enabling participants to ask questions in a REDs educational tool has the potential to more actively engage the participant in the learning experience, increasing learner motivation.

Irrespective of the delivery method, triathletes and coaches prefer content to be specific to triathlon, and to integrate expert advice with real‐life contextual experiences. A combination of expert and athlete delivery is most likely to be successful with real‐life athlete anecdotes supported by evidence‐based explanation and guidance. Our analysis of the data collected in this study revealed that athletes who had experienced REDs and experienced positive consequences of recovery had better ‘buy in’ and were more likely to modify their behaviour to manage REDs. This coincides with literature in which athletes who have experienced REDs describe not being able to fully comprehend the syndrome until they experienced it (Gillbanks et al. 2022a). Sharing athlete narratives has the potential to provide more relevant context, leading to higher engagement from the user. This aligns with educational literature, which found that learners are more engaged when educators share rich, authentic narratives (Miller‐Day et al. 2015). The narrative immersion model posits that narratives are a powerful tool for communication and persuasion, when used thoughtfully and ethically (Shaffer et al. 2018). If athletes and coaches feel more connected to content in the REDs education resource their motivation and learning engagement are more likely to improve.

Important challenges to address when developing REDs education are the normalisation of REDs symptoms and entrenched leanness culture that was revealed in both athletes and coaches during our analysis. Athlete descriptions of loss of menstruation being ‘normal’, interactions with health professionals telling them to lose weight, and the *‘push through mentality within triathlon*’, highlight some potential topics to cover for future educational interventions. Similar findings have been reported in other sports with athletes ignoring or downplaying symptoms such as amenorrhoea (Gillbanks et al. 2022a, 2022b; Langbein et al. 2021; O'Donnell et al. 2023). Research in running populations also describe unattainable body image ideals and coach power dynamics that perpetuate the leanness culture within the sport (Carson et al. 2021). Resistance to disrupting existing culture in triathlon may deter engagement with REDs education. Education approaches for female triathletes and their coaches need to navigate content sensitively to ensure that the push through mentality, normalisation of REDs symptoms and leanness culture is respectfully challenged.

This study considers the views and experiences of female triathletes and their coaches. It provides useful insight into opportunities and challenges to developing triathlon‐specific REDs education. We included athletes from a range of countries and different athlete calibres, although we interviewed a smaller number of elite than recreational athletes. We recognise that our recruitment approach likely captured athletes and coaches who are more interested in REDs education and this limits generalisability of our findings.

## 5 Conclusion

This analysis identified factors likely to influence engagement with a REDs education resource for female triathletes and their coaches. Participants in our study indicated strong capability and motivation to engage with REDs education. However, tensions between wanting to address REDs within the existing triathlon‐entrenched culture challenged engagement. There is an opportunity to improve female triathletes' understanding of REDs through the use of podcasts. Coaches might require a complementary program delivered in a more traditional learning format such as online modules or workshops. Both coaches and athletes expressed a desire for education to be triathlon specific, and a combination of content delivered by experts with real‐life narratives from other triathletes weaved throughout.

## Funding

The authors have nothing to report.

## Ethics Statement

University of Canberra Human and Research Ethics committee (13719).

## Conflicts of Interest

The authors declare no conflicts of interest.

## Supporting information


Supporting Information S1

## Data Availability

The data that support the findings of this study are available on request from the corresponding author. The data are not publicly available due to privacy or ethical restrictions.
