# Supplementary material for: Preferences of Female Triathletes and Their Coaches for Education on REDs: A Qualitative Study
Source: Eur J Sport Sci. 2026 Aug 14;26(9):e70240. doi: 10.1002/ejsc.70240 (PMC13476341; doi:10.1002/ejsc.70240)
Supplement: Supplementary file 1 [file EJSC-26-e70240-s001.docx]

**Supplementary material one:**

**TITLE OF THE STUDY:** Characterisation of female triathletes and coaches of female triathletes’ knowledge of REDs resources.

**Legend:** Relative Energy Deficiency in Sport (REDs). This is a Semi-structured interview guide used during the qualitative study of female triathletes and coaches of female triathletes. Questions focus on athlete and coach backgrounds (characterisation), current knowledge and experience of REDs, preferences for delivery style, previous education experiences and perception of culture relevant to REDs within triathlon

**Participant information:**

**Name:**

**Coach or athlete:**

**Age:**

**Date:**

**Interview location: Online**

Introduction:

Duration: The length of interview will be 30-60minutes

Primary goal: To understand what access you have to Relative Energy Deficiency in Sport (REDs) related information within the triathlon context and to understand how participants would like to receive information on the topic.

Preambles:

- Researcher background.
- Please understand that you can withdraw from this study at any time without any consequences and that you may decline to answer any question or stop the interview at any time.

Characterisation of participants:

- How long have you been training in triathlon and how did you get into the sport?

Athletes:

- At what level do you compete? Age group, professional (world champs, national, state etc).
- What distances do you compete at? (e.g short course, long course etc)
- Are you part of a squad environment where you train with a group in a regular basis or do train by yourself?
- Do you have a coach or coaches and if so, how long have you been working with them?

Coaches:

- How long have you been coaching and how did you get into the sport?
- What is your coaching accreditation status? Do you hold a TA coaching accreditation or other form of relevant qualifications (i.e. Grad. Cert., degree, etc.)?
- How many athletes do you coach and what level do the athletes you coach compete at?
- Do you identified yourself as a female/male/other?
- Do you coach female/male/other athletes? If a combination of them – how many of each?
- Do you coach in person/ online/ only group sessions or a combination??
- Could you briefly describe your philosophy to coaching?

REDs knowledge:

- What is your understanding of Relative Energy Deficiency in Sport (REDs)?
- When and where did you first hear the term REDs and what piqued your interest in the topic? (Trigger)
- Do you have any personal experience with REDs (either as an athlete or a coach of an athlete who has experienced it)?

Accessing resources:

- If you have, where have you gathered information about REDs from in the past?
- When accessing the information about REDs, how credible did you think the sources were and why?
- Based on your experience what do have you considered a reliable or valuable source of information on REDs?
- Who, in your opinion, is well-qualified to offer advice and insights on REDs?
- What qualities or expertise do you believe make someone a credible source?

Formal education resources:

- Have you had any formal education seminars or workshops in relation to triathlon?
- If yes, of the education resources been through your coaching pathways or through your triathlon organisation?
- If so, what did the program look like?
- What aspects did you find valuable about the resource?

Accessing education:

- How do you prefer to access information when it comes to topics related to triathlon?
- Have you experienced an education resource that you engaged with well? If so, what did you like about the education resource? What made it engaging?
- Are there education resources that you haven’t engaged with or completed? If so, what put you off from engaging with the resource?
- Can you please share how you might like to receive education on REDs or related material?
- For example, what medium you might find beneficial to access the education?
- Are there any specific resources or approaches you might find particularly beneficial when accessing education resources in triathlon?
- What info would you like to know about REDs?
- In a typical week how much time do you think you could dedicate to engaging with a resource directed to athletes and coaches on REDs related material?

REDs integration:

- How have you used the information you have learnt about REDs in your everyday coaching or athletic pursuits?
- Has your knowledge or experience of REDs affected the way you train? If so, how so?
- Can you please provide some specific examples?

Culture in Triathlon:

- What is your perspective of the knowledge/culture around REDs in triathlon?
- Do you think body weight is emphasised in triathlon? If so, what do you think contributes to that leanness culture?
- Do you think it can be improved? If so, how?
